# 固相萃取-超高效液相色谱-串联质谱法测定动物源食品中氯苯胺灵残留

**DOI:** 10.3724/SP.J.1123.2021.02009

**Published:** 2022-01-08

**Authors:** Lixia YANG, Xiaobei HUANG, Xike ZENG, Zi YI

**Affiliations:** 1.长沙市食品药品检验所, 湖南 长沙 410036; 1. Changsha Institute for Food and Drug Control, Changsha 410036, China; 2.国家酒类产品质量监督检验中心, 湖南 长沙 410036; 2. National Alcohol Products Quality Supervision and Inspection Center, Changsha 410036, China

**Keywords:** 超高效液相色谱-串联质谱, 固相萃取, 氯苯胺灵, 动物源食品, 残留, ultra-high performance liquid chromatography-tandem mass spectrometry (UHPLC-MS/MS), solid phase extraction (SPE), chlorpropham, animal derived foods, residues

## Abstract

建立了固相萃取-超高效液相色谱-串联质谱法(SPE-UHPLC-MS/MS)测定动物源食品中氯苯胺灵残留的分析方法。动物源食品经乙腈溶液提取、正己烷脱脂、ProElut PLS固相萃取柱富集净化,采用C18色谱柱进行分离,以0.2%(v/v)甲酸水溶液和乙腈为流动相进行梯度洗脱,采用电喷雾电离、正离子模式扫描,多反应监测模式(MRM)检测,基质匹配标准曲线外标法定量。研究通过对不同填料的固相萃取柱的考察,最终选择了ProElut PLS固相萃取柱,其能对动物源食品复杂基质中的氯苯胺灵进行有效富集和净化;通过考察3种规格的C18柱对氯苯胺灵的保留能力和出峰效果,选择了Agilent ZORBAX SB-C18(150 mm×2.1 mm, 5 μm)作为氯苯胺灵的分离色谱柱;通过考察氯苯胺灵在正负两种质谱电离模式下的响应情况,选择了正离子模式,确定氯苯胺灵一级质谱准分子离子和相应的碎片离子。结果表明:氯苯胺灵在0.5~100.0 μg/L范围内呈良好的线性关系,相关系数(*r*^2^)均不低于0.9929;方法定量限(LOQ)为3 μg/kg(*S/N*≥10);以猪肉、牛奶、牛肉、鸡肉、鸭肉、鸡蛋、鸡胗、鸭蛋、猪腰、猪肝、牛肝、羊肉、鸭胗共13种动物源食品为基质,在0.003、0.006、0.060 mg/kg添加水平下,氯苯胺灵的平均回收率为74.9%~97.6%,相对标准偏差(RSD)为2.9%~9.5%(*n*=6)。本方法前处理简便易行、基质干扰小、灵敏度高、准确性高,适用于多种动物源食品中氯苯胺灵的定性和定量检测。

## Abstract (en)

Chlorpropham is a plant growth regulator and a herbicide. It is commonly used in the post-harvest treatment of potato to inhibit germination. It can also be used for flower thinning and fruit thinning of fruit trees, and for controlling annual gramineous weeds and a few broad-leaved weeds. Improper or excessive use of chlorpropham in crop cultivation will affect the safety of animal-derived food and impair human health through the food chain and water cycle. Therefore, accurate quantification of chlorpropham is imperative for risk assessment and mitigating risks to food safety.

A method based on solid phase extraction and ultra-high performance liquid chromatography-tandem mass spectrometry (SPE-UHPLC-MS/MS) was established for the determination of chlorpropham in animal-derived food. First, the pretreatment conditions were optimized. To purify the samples and remove impurities, SPE column cartridges with different packing materials such as PXC, PXA, Florisil, and PLS were investigated. Based on the retention of chlorpropham, the ProElut PLS SPE column was selected as the pretreatment purification column. The washing solution and eluents were then optimized. When water was used as the washing solution, chlorpropham remained adsorbed on the SPE column and was not eluted along with other water-soluble substances. When the proportion of acetonitrile exceeded 40%, chlorpropham adsorbed on the filler of the SPE column could be gradually washed down. Acetonitrile-water solution(30:70, v/v) was used for washing the SPE column. The elution ability of seven eluents for chlorpropham on the SPE column was then investigated. Among them, pure methanol, pure acetonitrile, and 1% (v/v) formic acid-methanol showed better elution effect. Considering that acetonitrile was used in the sample extraction, it was chosen as the mobile phase eluent. Subsequently, the chromatographic conditions and MS parameters were optimized. By examining the ionization cracking of chlorpropham, the quasimolecular ions and corresponding fragmentations in the chlorpropham primary MS were determined. The separation effect of three C18 columns was investigated. Based on the retention ability and peak effect of chlorpropham on the column, the Agilent ZORBAX SB-C18 (150 mm×2.1 mm, 5 μm) column was used for chlorpropham separation. The response of chlorpropham in the positive and negative ionization modes was investigated and optimized. The results showed that the response was better in the positive ion mode than that in the negative ion mode. After optimizing the chromatographic conditions and MS parameters, the sensitivity of the method was improved. Finally, the analytes were separated on the Agilent ZORBAX SB-C18 (150 mm×2.1 mm, 5 μm) under a gradient elution program using acetonitrile and 0.2% (v/v) formic acid aqueous solution as the mobile phases. The analytes were detected in the multiple reaction monitoring (MRM) mode under positive electrospray ionization (ESI^+^) conditions.

The standard curve solutions were prepared using the matrix solution and quantified by the external standard method. The results showed a good linear relationship in the range of 0.5-100.0 μg/L, with correlation coefficients (*r*^2^) greater than 0.9929. The limit of quantification (LOQ) of this method was 3 μg/kg (*S/N* ≥ 10). At three spiked levels (0.003, 0.006, and 0.060 mg/kg) in 13 animal-derived foods (pork, milk, beef, chicken, duck, egg, chicken gizzard, duck egg, pork kidney, pork liver, beef liver, mutton, duck gizzard), the average recoveries were in the range of 74.9% to 97.6%, and the RSDs were in the range of 2.9% to 9.5% (*n*=6). Sixty batches of animal-derived food available on the market were analyzed by the developed method, and chlorpropham was not detected in any of these foods. The developed method is rapid, sensitive, and accurate, and it is suitable for the qualitative and quantitative detection of chlorpropham in a variety of animal-derived foods.

氯苯胺灵(chlorpropham,结构式见图1)既是植物生长调节剂又是除草剂,常用于马铃薯的采后处理,以抑制其发芽^[1,2]^,也可用于果树的疏花、疏果及防除一年生禾本科杂草和少数阔叶杂草^[3]^。按照我国农药规定,氯苯胺灵为低毒农药,但是氯苯胺灵在作物种植中的不当或过量使用,会随着食物链和水循环影响动物源食品安全以及人体健康^[4,5]^。因氯苯胺灵母体和代谢物3-氯苯胺灵具有严重的慢性风险,对消费者、非靶标节肢动物存在潜在内分泌干扰特性,欧盟委员会决定不再批准氯苯胺灵的使用^[6]^。目前,猪、牛、羊、鸡、鸭等动物饲养周期短,易导致药物在动物体内代谢不完全,存在药物残留风险。为降低食品安全风险,建立一种动物源食品中氯苯胺灵残留量的检测方法,可为氯苯胺灵的风险评估提供检测技术依据。

**图 1:**
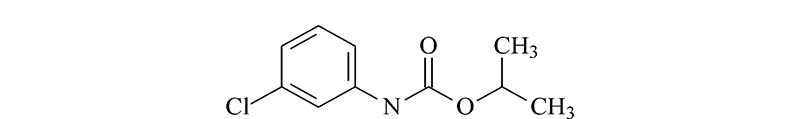
氯苯胺灵的分子结构式

根据GB 2763-2019《食品中农药残留最大残留量》^[7]^,氯苯胺灵在牛肉中的限量值为0.1 mg/kg、牛内脏和生乳限量值均为0.01 mg/kg,检测方法为GB 19650-2006《动物肌肉中478种农药及相关化学品残留量的测定》^[8]^和GB/T 23210-2008《牛奶和奶粉中511种农药及相关化学品残留量的测定》^[9]^;氯苯胺灵在马铃薯中的限量值为30 mg/kg,检测方法为GB 23200.9-2016《粮谷中475种农药及相关化学品残留量的测定》^[10]^、GB 23200.113-2018《植物源性食品中208种农药及其代谢物残留量的测定》^[11]^,采用的均为气相色谱-质谱法。文献报道氯苯胺灵的检测方法有反相高效液相色谱法^[12]^、气相色谱法、液相色谱法^[13]^、气相色谱-质谱法^[14]^等。现有文献大多针对植物源性样品,对动物源性食品中氯苯胺灵的研究较少。液相色谱-串联质谱法与气相色谱-质谱法相比,具有较高的灵敏度,抗干扰能力强,检测时间更短,检测效率更高,更加适用于大批量样品的检测。本文结合氯苯胺灵的物理和化学性质、固相萃取的净化优势和超高效液相色谱-串联质谱的高灵敏特性,优化前处理过程和色谱-质谱条件,开发出了一种固相萃取-超高效液相色谱-串联质谱法(SPE-UHPLC-MS/MS)来测定不同动物源食品中氯苯胺灵残留。

## 1 实验部分

### 1.1 仪器与试剂

AB SCIEX QTRAP 5500^+^型超高效液相色谱-串联三重四极杆质谱联用仪(美国AB SCIEX公司), SF-16R型高速冷冻离心机(美国Thermo Fisher Scientific公司), Direct-Q^®^ 8 UV-R型超纯水机(美国Millipore公司), MV5全自动高通量平行浓缩仪(莱伯泰科有限公司), HES-24B真空固相萃取装置(天津市恒奥科技发展有限公司), KQ5200DE型数控超声波清洗器(昆山市超声仪器有限公司), CM-1000型振荡混匀器(日本EYELA公司), GM200刀式研磨仪(德国Retsch(莱驰)), ProElut PLS固相萃取柱(200 mg/6 mL)(上海迪柯马分析技术有限公司)。

氯苯胺灵标准溶液(1000 mg/L)购自农业部环境质量监督检验测试中心;甲醇(色谱纯)和乙腈(色谱纯)购于德国Merck KGa A公司;正己烷(色谱纯)购于美国Tedia公司;甲酸(色谱纯)购于aladdin公司;无水硫酸钠(AR纯)和氯化钠(AR纯)购于国药集团化学试剂有限公司,0.22 μm聚四氟乙烯滤膜(PTFE)购自希波氏公司。

13种动物源食品(猪肉、牛奶、牛肉、鸡肉、鸭肉、鸡蛋、鸡胗、鸭蛋、猪腰、猪肝、牛肝、羊肉、鸭胗)均购自当地市场。

### 1.2 标准溶液的配制

标准储备液:准确吸取1 mL氯苯胺灵标准溶液(1000 mg/L),用甲醇稀释定容至25 mL,配制成40 mg/L的氯苯胺灵标准储备液转移至棕色玻璃瓶中,于0~4 ℃避光储存。

标准工作液:准确吸取氯苯胺灵标准储备液适量,用甲醇稀释,配制成1.0 mg/L的标准工作液,于0~4 ℃避光储存,备用。临用时,用空白基质溶液逐级稀释成适当浓度,待测。

### 1.3 样品制备

1.3.1 样品预处理

牛、羊、猪、鸭、鸡等畜禽类样品取可食肌肉组织、内脏约300 g,切碎后经刀式研磨仪充分搅碎混匀,装入洁净容器内,密封,于-18 ℃以下冷冻保存。牛奶样品取300 g,混匀,装入洁净容器内,密封,于0~4 ℃冷藏保存。蛋类样品取10枚,去壳后经刀式研磨仪充分搅碎混匀,装入洁净容器内,密封,于0~4 ℃冷藏保存。

1.3.2 样品前处理

称取均质样品3.0 g(精确至0.01 g)于50 mL具塞塑料离心管中,静置使其恢复至室温;用移液管准确移取10 mL乙腈加入至样品中,超声5 min,均质15 min;加入约2 g氯化钠和约3 g无水硫酸钠,均质3 min; 10000 r/min离心3 min;取上清液7.0 mL于10 mL塑料离心管中;加入用乙腈饱和过的正己烷2 mL,均质2 min, 10000 r/min离心2 min,去除正己烷层;准确移取5 mL提取液于带刻度的具塞离心管中,45 ℃氮吹浓缩至约0.5 mL,用超纯水定容至5 mL,待进一步净化。

用5 mL甲醇和5 mL水活化ProElut PLS固相萃取柱,上样,用5 mL 30%乙腈水溶液淋洗除杂后抽干小柱,用8 mL乙腈洗脱,洗脱液于45 ℃氮吹至约0.5 mL,用乙腈-0.2%(v/v)甲酸水溶液(1∶1, v/v)定容至2 mL,过0.22 μm PTFE滤膜,待测。

### 1.4 色谱-质谱条件

色谱条件 色谱柱:Agilent ZORBAX SB-C18(150 mm×2.1 mm, 5 μm);柱温:40 ℃;流速:0.5 mL/min;流动相:A为0.2%(v/v)甲酸-水溶液,B为乙腈。流动相梯度洗脱程序:0~1.00 min, 15%B; 1.00~5.00 min, 15%B~90%B; 5.00~8.00 min, 90%B; 8.00~8.01 min, 90%B~15%B; 8.01~10.0 min, 15%B。进样体积:5 μL。

质谱条件 离子源:电喷雾电离(ESI)源;扫描方式:正离子扫描;离子源温度(TEM): 550 ℃;雾化气(Gas 1)压力:379 kPa(55 psi);辅助气(Gas 2)压力:379 kPa(55 psi);离子喷雾电压(IS):+5500 V;气帘气(CUR)压力:241 kPa(35 psi);检测方式:多反应监测(MRM)模式。氯苯胺灵在正离子模式下的质谱分析参数见表1。

**表 1:** 氯苯胺灵在正离子模式下的质谱分析参数

Analyte	Precursor ion (m/z)	Product ion (m/z)	DP/V	CE/eV
Chlorpropham	213.9	172.0^*^	73	15
		154.1	73	24

* Quantitative ion. DP: declustering potential; CE: collision energy.

## 2 结果与讨论

### 2.1 色谱条件和质谱条件的优化

2.1.1 色谱柱的选择

氯苯胺灵为弱极性化合物,液相色谱方法常用的色谱柱为C18柱^[15,16]^,本方法比较了3种规格的C18色谱柱的分离效果(见图2)。本方法以猪肉样品为研究对象,在流动相均采用0.2%(v/v)甲酸水溶液-乙腈的梯度洗脱条件下,比较了Agilent ZORBAX SB-C18(150 mm×2.1 mm, 5 μm)、Agilent ZORBAX SB-C18(100 mm×2.1 mm, 5 μm)和Agilent Eclipse Plus C18(150 mm×2.1 mm, 5 μm)3种色谱柱对氯苯胺灵的保留能力和出峰效果。结果表明,100 mm柱与150 mm柱比较,保留时间出现前移,但峰形有明显的拖尾;EclipsePlus C18柱与ZORBAX SB-C18相比,采用ZORBAX SB色谱柱时,氯苯胺灵的峰形更好,更对称。因此,选用Agilent ZORBAX SB-C18(150 mm×2.1 mm, 5 μm)作为氯苯胺灵的分离色谱柱。

**图 2:**
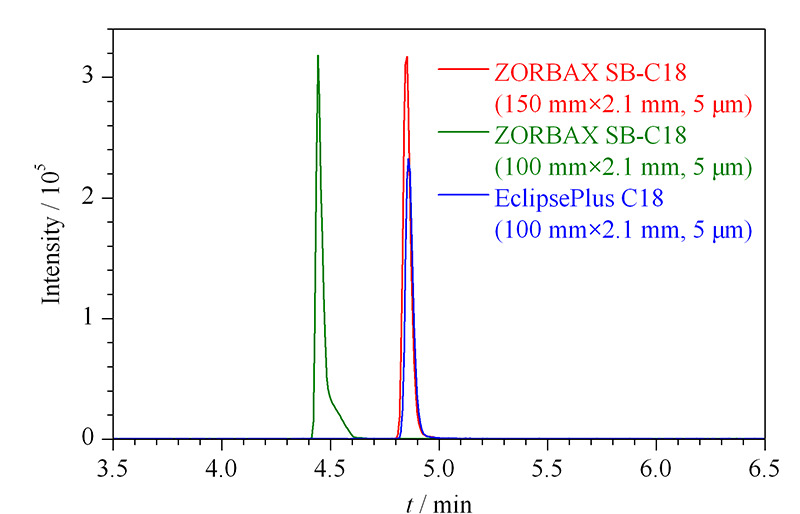
采用不同色谱柱时氯苯胺灵的色谱图

2.1.2 质谱条件的优化

本实验分别考察了氯苯胺灵在正负两种质谱电离模式条件下的响应情况。取1.0 mg/L的氯苯胺灵工作液,分别用正离子模式和负离子模式进行条件优化。结果表明,正离子模式的响应要明显优于负离子模式。氯苯胺灵分子结构中带有-NH,可能容易加合氢离子形成带正电荷的离子,提高离子化效率,故氯苯胺灵在正离子环境中响应更高。

在针泵连续进样方式下,采取Q1 Scan全扫描模式对目标物进行全扫,得到母离子(*m/z* 213.9),采取Product Ion Scan(MS2)模式对子离子进行扫描,得到相对丰度较高的特征碎片离子:*m/z* 172.0和*m/z* 154.1。对特征离子对的仪器参数(去簇电压和碰撞能)进一步优化,得到最优化的分析参数。

2.1.3 流动相的选择

在正离子模式下,考察了3种流动相体系:0.1%(v/v)甲酸水溶液-乙腈流动相体系、0.2%(v/v)甲酸水溶液-乙腈流动相体系和水与乙腈流动相体系(见图3)。结果表明,采用甲酸水-乙腈流动相体系时,氯苯胺灵的响应明显高于水-乙腈流动相体系,并且随着酸性的提高,响应更好,峰形更对称且尖锐。因此,实验选择0.2%(v/v)甲酸水溶液-乙腈作流动相。

**图 3:**
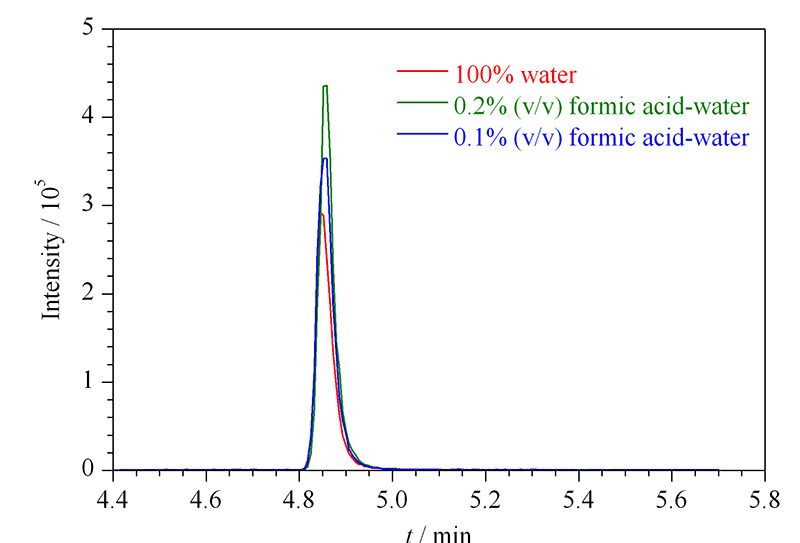
采用不同流动相时氯苯胺灵的色谱图

### 2.2 前处理条件的优化

2.2.1 提取溶剂的选择

氯苯胺灵难溶于水,易溶于甲醇、乙腈等有机溶剂,对植物基质进行分析时常用正己烷、乙腈等有机溶剂进行提取^[11,17]^。考虑到本方法检测对象为动物源食品,其成分较为复杂,蛋白和油脂含量都比较高,故使用肉类提取常用溶剂乙腈进行提取,并使用正己烷对提取液进行除油处理。

2.2.2 固相萃取柱的选择

各种SPE柱对样品的净化效果不同,本研究比较了阳离子交换SPE柱ProElut PXC(60 mg/3 mL)、阴离子交换SPE柱ProElut PXA(60 mg/3 mL)、硅酸镁SPE柱ProElut Florisil(200 mg/3 mL)、亲水亲脂平衡SPE柱ProElut PLS(200 mg/6 mL)4种SPE柱对氯苯胺灵的柱保留量。采用5 mL 0.1 mg/L的氯苯胺灵标准溶液过柱,测定流出液中氯苯胺灵的含量,计算4种SPE柱的柱保留量,其柱保留量百分比分别为:PLS 98.3%、PXA 85.3%、PXC 88.7%和Florisil 60.2%。结果表明,PLS SPE柱的保留能力较强,可提高方法的最终回收效率。

2.2.3 淋洗液和洗脱液的选择

淋洗液:以体积比为0∶100、10∶90、20∶80、30∶70、40∶60、50∶50、60∶40、70∶30、80∶20、90∶10、100∶0的乙腈-水溶液作为淋洗液,收集滤液,采用优化好的色谱-质谱条件测定。结果显示:当淋洗液乙腈-水溶液有机相比例达到≥40%时,滤液中可检出氯苯胺灵,且含量随着有机相比例升高而增大(见图4)。为有效去除蛋白、油脂等杂质,又能获得更好的回收率,本实验选用乙腈-水溶液(30∶70, v/v)作为固相萃取淋洗液。

**图 4:**
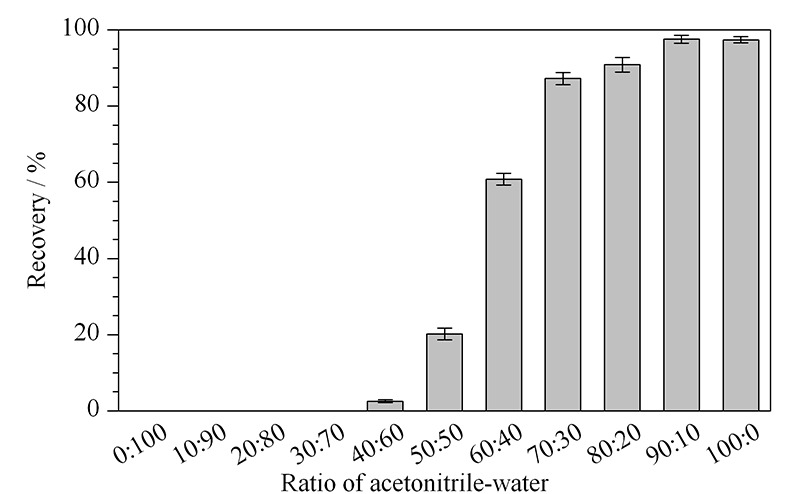
不同体积比例的乙腈-水溶液对氯苯胺灵的淋洗能力(*n*=3)

洗脱液:比较了100%甲醇、100%乙腈、1%甲酸-甲醇、90%甲基叔丁基醚-乙腈、5%氨水-甲醇、100%乙酸乙酯、90%乙酸乙酯-甲醇7种常用洗脱溶剂对氯苯胺灵在ProElut PLS SPE柱上的洗脱能力。同时用以上7种溶剂配制标准溶液。洗脱能力采用洗脱后测定的峰面积(*A*_X_)与配制的标准溶液峰面积(*A*_0_)之比评价(洗脱能力=*A*_X_/*A*_0_×100%),结果依次为97.8%、98.5%、95.5%、89.3%、87.1%、68.3%、69.5%,表明用100%甲醇、100%乙腈、1%甲酸-甲醇3种溶液洗脱效果较好,考虑到样品提取使用的是乙腈,本实验选用乙腈作为流动相洗脱液。

### 2.3 基质效应(ME)评价

基质效应是指样品中除分析物以外的组分在分析过程中对目标物有显著的干扰,从而影响分析结果的准确性。本实验绘制基质匹配标准溶液曲线和溶剂标准溶液曲线,通过两个标准曲线斜率的比值对基质效应进行评估^[18]^。基质效应=基质匹配标准溶液曲线斜率/溶剂标准溶液曲线斜率×100%。当基质效应值在85%~115%之间时,说明无明显基质效应。

本工作用猪肉、牛奶、牛肉、鸡肉、鸭肉、鸡蛋、鸡胗等空白样品基质提取液和溶剂溶液(乙腈-0.2%(v/v)甲酸水溶液(1∶1, v/v))分别配制成0.5、1.0、2.25、4.5、9.0、45.0、100.0 μg/L系列标准溶液,按照优化好的色谱-质谱条件进行分析,对氯苯胺灵的峰面积(*y*)与质量浓度(*x*, μg/L)进行线性回归,绘制标准曲线,计算基质效应,结果表明:13种样品的基质效应值在90.9%~106.6%之间(见表2),基质增强或减弱效应不明显,但由于各基质成分复杂且存在一定差异性,为了检测的准确性,所有样品检测均用空白基质匹配溶液配制标准溶液进行样品检测。

**表 2:** 不同基质中氯苯胺灵的线性方程、线性范围、相关系数(*r*^2^)和基质效应

Matrix	Linear equation	r^2^	ME/%
Pork	y=3004.0968x+517.9608	0.9964	99.2
Milk	y=3976.8934x+1868.2353	0.9975	98.4
Beef	y=3565.0949x+1298.4540	0.9984	95.1
Chicken	y=3488.3735x+997.4987	0.9931	98.9
Duck	y=3105.5786x+1573.7086	0.9971	90.9
Egg	y=3505.5015x+1046.4965	0.9991	101.7
Chicken gizzard	y=5389.5996x+974.5213	0.9978	92.3
Duck egg	y=7567.7369x+1932.6471	0.9929	99.9
Pork kidney	y=6865.0184x+3035.3652	0.9951	97.6
Pork liver	y=5115.4132x+2716.0322	0.9998	99.23
Beef liver	y=6728.9065x+915.4150	0.9992	104.1
Mutton	y=6129.0758x+1904.7427	0.9992	106.6
Duck gizzard	y=6075.7672x+168.6431	0.9997	96.7

* Linear range: 0.5-100 μg/L. *y*: peak area; *x*: mass concentration, μg/L.

### 2.4 线性范围和定量限

表2数据同时表明,猪肉、牛奶、牛肉、鸡肉、鸭肉、鸡蛋、鸡胗、鸭蛋、猪腰、猪肝、牛肝、羊肉、鸭胗13种样品基质中氯苯胺灵在0.5~100 μg/L范围内的线性关系均良好,其线性相关系数均不低于0.9929。本实验根据所测基质样品溶液信噪比(*S/N*)≥10计算得到该方法检测氯苯胺灵的定量限(LOQ)为0.003 mg/kg,与文献报道和国标方法相比具有更高的灵敏度:文献^[17]^的方法定量限为0.0067 mg/kg,GB 19650-2006^[8]^的方法定量限为0.083 mg/kg,GB/T 23210-2008^[9]^的方法定量限为0.028 mg/kg(牛奶基质)和0.139 mg/kg(奶粉基质)。

### 2.5 准确度和精密度

通过对空白样品进行加标回收,考察本方法的准确度与精密度。按照优化后的提取和净化步骤,在上述13种基质样品中分别添加0.003、0.006、0.060 mg/kg 3个水平的氯苯胺灵标准溶液进行加标试验,每个加标水平做6个平行(见表3),不同基质加标水平为3 μg/kg时的色谱图见图5。结果显示,在低、中、高3个加标水平下,不同基质样品中氯苯胺灵的平均回收率为74.9%~97.6%,相对标准偏差为2.9%~9.5%,符合GB/T 27404-2008《实验室质量控制规范食品理化检测》^[19]^规定的要求,可用于动物源食品中氯苯胺灵的定量检测。

**表 3:** 不同基质中氯苯胺灵的加标回收率和精密度(*n*=6)

Matrix	0.003 mg/kg				0.006 mg/kg				0.060 mg/kg		
	Recovery/%	RSD/%			Recovery/%	RSD/%			Recovery/%	RSD/%	
Pork	84.5		6.0		92.5		6.6		92.1		3.4
Milk	79.2		7.2		85.6		6.0		80.7		5.8
Beef	87.7		5.5		88.2		8.5		91.9		5.1
Chicken	77.2		6.7		82.8		4.9		80.2		6.0
Duck	79.6		6.7		79.6		3.4		83.3		5.7
Egg	92.8		6.6		91.7		6.9		89.2		3.3
Chicken gizzard	76.9		5.7		83.8		6.3		76.3		5.4
Duck egg	74.9		4.1		77.3		9.5		80.1		8.1
Pork kidney	79.1		8.7		85.0		6.1		86.4		8.2
Pork liver	83.3		7.8		79.9		9.0		90.3		4.2
Beef liver	90.1		4.6		97.6		7.1		87.9		7.0
Mutton	94.5		7.0		97.2		6.4		89.3		4.4
Duck gizzard	91.1		5.1		95.6		5.1		90.4		5.7

**图 5:**
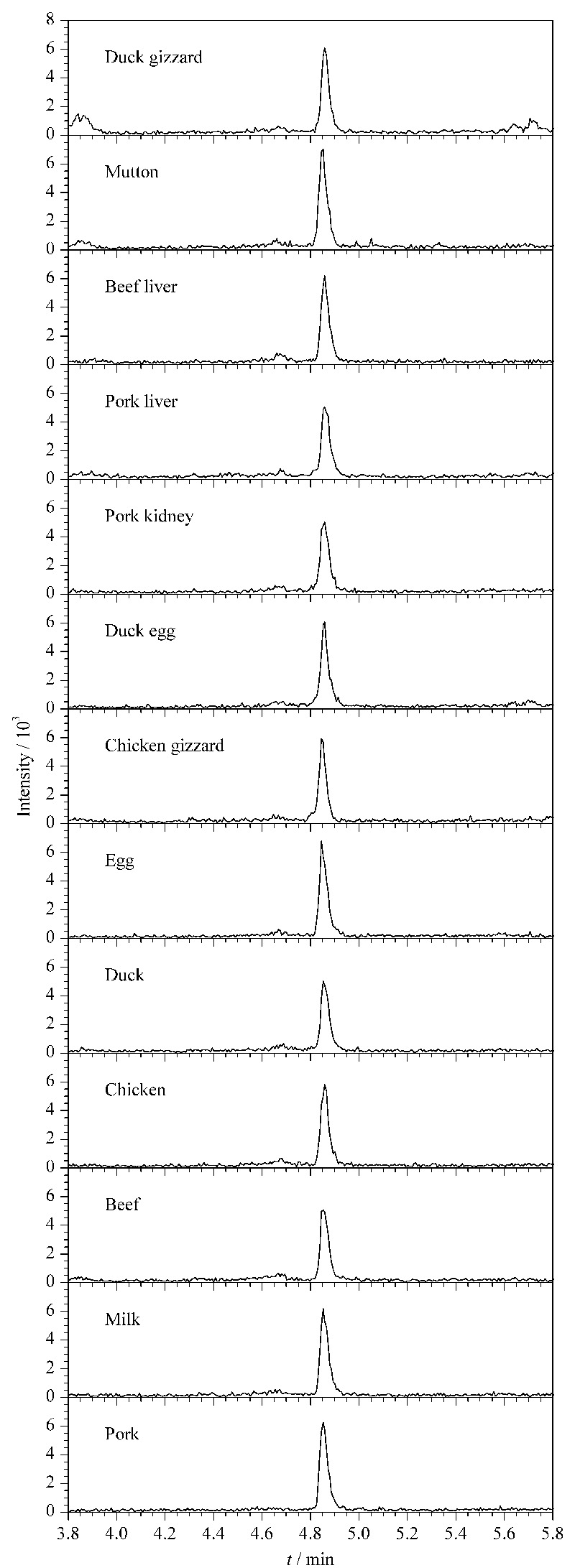
不同基质溶液加标样品中氯苯胺灵(3 μg/kg)的色谱图

### 2.6 实际样品的测定

利用本方法对市售的上述13种动物源食品共计60批次样品进行氯苯胺灵残留量检测,均未检出氯苯胺灵。

## 3 结论

本文通过优化色谱-质谱条件,采用乙腈提取,选择ProElut PLS SPE柱,优化 SPE淋洗液、洗脱液成分,以空白基质匹配溶液配制标准曲线,建立了固相萃取-超高效液相色谱-串联质谱法测定动物源食品中氯苯胺灵残留的检测方法。该方法前处理简便易行,基质干扰小,灵敏度高,线性关系、准确度和精密度满足方法学要求,适用于多种动物源食品中氯苯胺灵的定性和定量检测。
